# Emotional Exhaustion in Housewives and Alzheimer Patients’ Caregivers: Its Effects on Chronic Diseases, Somatic Symptoms and Social Dysfunction

**DOI:** 10.3390/ijerph16183250

**Published:** 2019-09-04

**Authors:** Alina de las Mercedes Campos-Puente, María Luisa Avargues-Navarro, Mercedes Borda-Mas, Milagrosa Sánchez-Martín, José M. Aguilar-Parra, Rubén Trigueros

**Affiliations:** 1 Research Directorate, Inca Garcilaso de la Vega University, Cercado de Lima 7476, Peru; 2 Department of Personality, Evaluation and Psychological Treatment, CTS-432 Research Team, University of Seville, 41004 Seville, Spain; 3 Department of Psychology, University of Loyola Andalusia, 41014 Seville, Spain; 4 Department of Psychology, Hum-878 Research Team, Health Research Centre, University of Almeria, 04120 Almeria, Spain

**Keywords:** emotional exhaustion, chronic diseases, somatic symptoms, housewives, caregiver housewives, main caregivers of Alzheimer’s patients

## Abstract

Background: Emotional exhaustion causes adverse effects in those who suffer from it. Housewives are not excluded. Domestic and care chores, which are considered to be sources of stress, increase when taking on the role of caregiver for a family member with Alzheimer’s disease. Objective: To analyse the influence of emotional exhaustion, somatic symptoms and social dysfunction, based on the activity they carry out. Methodology: Cross-sectional survey. 193 women participated, of which: housewives (HWs) (n = 97), and Alzheimer’s patient caregiver-housewives (CHWs) (n = 96). The evaluation tools were: sociodemographic/working data questionnaire (ad hoc), Maslach Burnout Inventory (MBI) and Goldberg General Health Questionnaire (GHQ-28). Results: High rates of emotional exhaustion are observed, as well as an existing positive link between chronic diseases, somatic symptoms and social dysfunction. The structural model indicates that emotional exhaustion predicts the amount and extent of diseases, somatic symptoms and social dysfunction. The influence is higher in CHWs. Limitations: Sample procedure implemented at convenience; the variable of the grade of dependence of the Alzheimer’s patient caregiver was not included in the study. Conclusions: The domestic and care chores that HWs and CHWs carry out affect their health. Hence the need to develop psychoeducative programmes that are adapted to the particular needs of these women and focused on the different areas of their everyday lives.

## 1. Introduction

Burnout, as a “three-dimensional syndrome characterised by emotional exhaustion, depersonalisation and reduced personal accomplishment” (Maslach and Jackson, 1981), has been an object of study of many investigations over the last few decades, due to its increasing importance and consequences. This syndrome is considered to be an important risk factor for both physical and mental health, with consequences in the sufferer’s family and working life. Out of the three dimensions of burnout, emotional exhaustion is the basic individual stress component of the syndrome [1].

Maslach and Leiter [2] define exhaustion as feelings of excessive physical and emotional demands. They are the result of daily contact with the patients they provide service to, which entails a state of exhaustion that drains their personal resources.

Emotional exhaustion is considered by different authors as the central axis of the syndrome [3,4,5]. For Maslach and Leiter [6], it is the most predictive dimension of the stress-linked health results. The overload produced by working conditions hinders rest, recovery and the restoration of balance. 

There are very few studies that have been carried out with housewives, despite the fact that, traditionally, it is they who take on domestic and caregiver roles. This unpaid and socially undervalued task favours the development of stress, with a higher impact on those who assume the role of primary caregiver of the Alzheimer’s patient, without previous training or experience [7]. This has a high-level overload impact that affects the perception of one’s health condition [8] and psychosocial balance [9].

Along these lines, in a study of Campos-Puente [10], which was carried out with housewives and Alzheimer’s patient caregiver housewives, we can observe a higher level of emotional exhaustion in comparison with the other dimensions of the burnout syndrome. Equally, different studies report on the effects on health [11], expressed in the amount of pathologies [12]. They often point at exhaustion, physical symptoms, back pain, headaches, myalgia [13], as well as multiple pathologies, mainly osteoarticular-related [14], alterations of the immunological system [15], cardiovascular system and metabolic changes [16].

On the other hand, different studies make reference to the existence of somatic symptoms that have no organic cause, nor clinical explanation [17], which reflect emotional complications in vital family and social situations [18], and which have a higher prevalence in women [19]. Additionally, they point out slow-progressing and long-lasting chronic diseases (more than three months), with no initial symptoms, that could be prevented and/or controlled, but that in some cases have no cure [20]. Caregivers sometimes suffer from various medical pathologies. Hence the need to develop actions at a psychosocial level, due to its impact on the vulnerability to illness [21].

Alzheimer’s disease as suffered by the patient is considered to be an added psychosomatic risk factor for the family caregivers. This disease has adverse effects, not only for those who suffer it, but it also debilitates family members [22], affecting the caregiver’s health [23] as a result of its physical and emotional impact. 

As for social dysfunction, caregivers of Alzheimer’s patients adopt a self-destructive attitude, they lose their identity and the prospect of a future in their own lives [24] and show a tendency to isolate themselves, with a lack of interest in social interaction. At home, they display irritability, which has a detrimental effect on personal relations. It also contributes to the appearance, increase or decrease of behavioural and psychological symptoms of dementia in the ill family member, as they depend on the quality of the interaction with their caregivers [25].

From this point of view, inadequate behaviour is observed in the displays of affection within the family, which hinders mutual support in the face of the reality they have to deal with, conflict management and family decision making. Likewise, dysfunctional coping mechanisms are linked to a higher subjective load, regardless of the caregiver’s objective load suffered, gender or level of kinship [26]. 

At present, women stand out performing professionally in different areas of the labour market. However, in Spain, as in other countries of the European Union, their participation is lower than that of men. This situation derives from certain differences referring to the type of employment they perform, the qualification in the activity, among other reasons, which give rise to an “unequal gender salary gap” [27]. An important part of the population of women work as housewives, carrying out domestic work and care within their family environment, without remuneration, for long hours, with poor social support, which is aggravated when they also care for a family member with Alzheimer’s disease, abruptly limiting their levels of welfare and adequate enjoyment of their social life, affecting the general deterioration of their health. This is a latent and current problem that deserves to be studied, because it is a work that arises from the needs observed in clinical practice in this population.

Taking all the foregoing into account, the general objective of this study is to find out the influence that emotional exhaustion has on chronic diseases, somatic symptoms and social dysfunction in housewives and Alzheimer’s patient caregiver housewives.

The specific objectives are: (1) to determine the extent to which emotional exhaustion affects chronic diseases, somatic symptoms and social dysfunction in the participants; (2) to find out whether there is a link based on the type of activity that they carry out as housewives or caregiver housewives for the Alzheimer’s patient. 

## 2. Methods

### 2.1. Participants

The sample was made up of 193 women, divided into: housewives (HWs) (n = 97) and Alzheimer’s patient caregiver housewives (CHWs) (n = 96), all belonging to the *Confederación Andaluza de Asociaciones de Familiares de Enfermos de Alzheimer* (Andalusian Confederation of Alzheimer’s Patients’ Families Association) and Women’s Associations of Seville, in Spain. They were between 20 and 80 years old (Mean = 49, Standard Deviation = 11.31), mainly holding primary (59.6%) and secondary education (20.2%). Regarding their marital status, 78% lived with their partners, of which 87.6% had children, and only 74.1% of those lived with them. The Alzheimer’s patients ranged in age from 67 to 85. The length of time they were in care ranged from 2 years to 14 years.

These women voluntarily participated once a week in the different workshops organised by the association. 

The inclusion criteria for the two groups (housewives and caregiver housewives) were: to be a woman, to be older than 20 years of age, to only work at home and for this activity to be unpaid. 

In addition, for the caregivers it was essential to be related to the patient and to have been caring for the patient for more than two years.

### 2.2. Instruments

Sociodemographic data and health indicators questionnaire (ad hoc). It consists of 10 open and close questions related to sociodemographic variables (marital status, education, children, number of children with whom they live), characteristics of the activity they carry out and health indicators (whether they suffer from chronic diseases, number of chronic diseases suffered, type of medical pathologies, number of months/years of medical treatment).

Maslach Burnout Inventory (MBI; [4]) with the Spanish adaptation by Seisdedos [27]. It consists of 22 items related to feelings and attitudes at work, and towards people who receive their services. The answers are based on a Likert scale from 0 (never) to 6 (everyday). It was produced following the three-dimensional structure: emotional exhaustion (EE), depersonalisation (DP) and personal accomplishment (PA) that conceptualises the burnout syndrome. For this study, we have only used the emotional exhaustion dimension (EE), composed of nine items (1-2-3-6-8-13-14-16-20). In order to classify participants into high (>24), average (15–24) or low (<15) scores in this dimension, we used the validation and reliability criteria proven by the authors for the Spanish population sample (n = 1.138) with an internal reliability and consistency of 0.75 and 0.90 [28].

General Health Questionnaire GHQ-28 [29], Spanish version [30]. It consists of 28 items, related to symptoms or behaviour experienced, divided into four subscales: A (somatic symptoms), B (anxiety and insomnia), C (social dysfunction) and D (depression). Each item has four possible answers, and the score scales go from “less than usual” in columns 1 and 2, with a value of “0”, to “much more than usual”, in columns 3 and 4, with a value of “1”. For this study, only subscales A (somatic symptoms) and C (social dysfunction) were used. The validity and reliability index referred to by the authors are satisfactory at 0.67 and 0.76, respectively. 

### 2.3. Procedure

Prior to commencing the study, people in charge of different organisations were contacted in order to access the sample population. Therefore, the convenience sampling method was used, based on accessibility. Once the association’s consent was obtained and the women who wanted to voluntarily participate in the study were selected, one day of the week was selected to summon all the participants to fill out all the questionnaires, which were answered anonymously. One member of the investigation group provided the informed consent form and the questionnaire to each participant. The participants answered anonymously and all ethical procedures were respected in accordance with the recommendations of the American Psychological Association. Furthermore, the data were obtained in strict adherence to current privacy and data protection laws and guidelines from the Helsinki Declaration.

### 2.4. Data Analysis

In this study, we carried out descriptive statistical analyses, bivariate correlations and reliability analyses using the statistic software SPSS v.24. Moreover, structural equation modelling (SEM) was carried out using AMOS v.21 [31] in order to test the relations established in the hypothesised model, a MANOVA and a univariate analysis.

**Figure 1:**
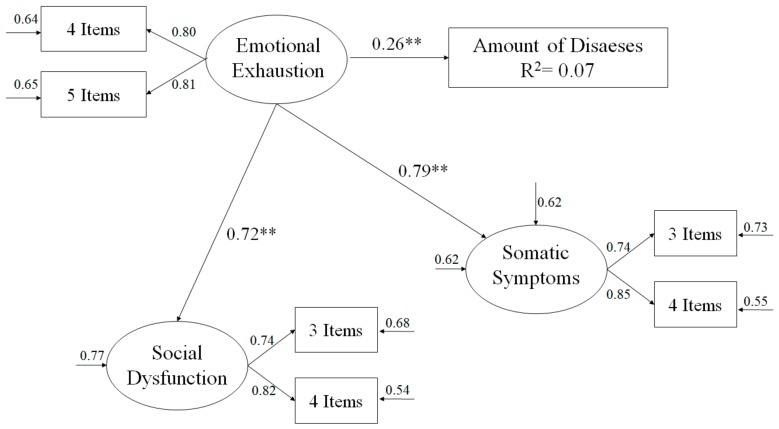
Structural equation model shows the links between emotional exhaustion, the number of diseases, social dysfunction and somatic symptoms. All parameters are standardised and are statistically significant. Explained variances are shown above the small arrows. Note: *** *p* < 0.001.

Given that Mardia’s rate was high (25.07) for the analysed hypothesised model (see Figure 1), the estimation method of maximum authenticity was used, together with the bootstrapping procedure. Estimators were not affected by the lack of normality and were therefore considered strong [32]. With the aim of accepting or rejecting the tested models and the confirmatory factory analysis (CFA), we used a series of adjustment indexes [33]: chi-square value divided by the degree of freedom (*χ*^2^*/df*); CFI (Comparative Fit Index); IFI (Incremental Fit Index); RMSEA (Root Mean Square Error of Approximation) together with its confidence interval (CI) to 90% and SRMR (Standardised Root Mean Square Residual). Given that the *χ*^2^ is very sensitive to the sample size [34], *χ*^2^*/df* was used, considering as acceptable those values that were below 5 [33]. Incremental indices (CFI and IFI) displayed a good adjustment with 0.90 values or above [35], while the error indices (RMSEA and SRMR) were considered acceptable with values that were equal to or below 0.08 [36].

## 3. Results

### 3.1. Preliminary Analysis

Descriptive statistics and the bivariate correlations through Cronbach’s α between the study’s variables can be observed in Table 1. 

The correlation analyses showed a positive relation amongst all factors. In this manner, the amount of diseases showed a positive relation with emotional exhaustion, somatic symptoms and social dysfunction. As for emotional exhaustion, it showed a positive relation with somatic symptoms and social dysfunction. Lastly, social dysfunction showed a positive relation with somatic symptoms.

### 3.2. Structural Equation Modelling

Before testing the hypothesised model and checking the predictive relations between the dimensions studied, the two-step method suggested by Anderson and Gerbing [37] was followed. Therefore, a CFA was initially carried out without restrictions in the model. The second step was to test the SEM and to analyse the existing links between the variables that belong to the model suggested. In order to achieve this, a reduction in the number of latent variables per factor was made, which is particularly relevant when the sample is not too large compared to the number of variables of the model [38,39]. This reduction could then be achieved through the combination of paired items. In this manner, half of the items on the first scale were averaged together in order to form the first block of items, the second half of items were averaged together to form the second block of items, and so on. This proposal is highly reliable, as they tend to be distributed normally, and it also halves the ratio of the variables in the model.

Even so, the CFA’s adjustment index was: *χ*^2^ (9, N = 193) = 24.39, *χ*^2^*/df* = 2.71, *p* < 0.05, IFI = 0.98, CFI = 0.98, RMSEA = 0.054 (Interval Confidence 90% = 0.045–0.071), SRMR = 0.042. The hypothesised predictive relations model (Figure 1) shows that the adjustment indexes were valid: χ^2^ (12, N = 193) = 53.72, *χ*^2^*/df* = 4.48, *p* < 0.001, IFI = 0.92, CFI = 0.92, RMSEA = 0.079 (IC 90% = 0.065–0.090), SRMR = 0.059. These results correspond to the established parameters, and therefore we can accept the suggested model as valid [33]. Equally, the contribution that each factor made to the prediction of other variables was examined through the standardised regression weight.

In Figure 1 we can observe how emotional exhaustion positively predicted the amount of diseases, as well as social dysfunction and somatic symptoms.

MANOVA’s interferential analysis showed the existence of important differences between housewives and caregiver housewives in relation to the whole of the three variables of emotional exhaustion, somatic systems and social dysfunction, showing the following adjustment indices: *p* = 0.001, F (189) = 26.46, Wilks’ lambda *η*^2^ = 0.704; *R*^2^ = 0.30. We can observe that it can affect them in different ways depending on the activity these women do.

Univariately, we can observe how meaningful differences exist between Alzheimer patient caregiver housewives and housewives, as far as emotional exhaustion is concerned, with the following adjustment indices: *p* = 0.001, F (189) = 38.50; *η^2^* = 0.17; *R*^2^ = 0.16. Regarding somatic symptoms, the adjustment indices were: *p* = 0.000, F (72.05) < 1; *η*^2^ = 0.27; *R*^2^ = 0.27, and lastly, for social dysfunction, adjustment indices were: *p* = 0.000, F (189) = 40.04; *η*^2^ = 0.17; *R*^2^ = 0.17. From this point of view, we can observe that taking on the role of the caregiver impacts emotional exhaustion, somatic symptoms and social dysfunction. 

## 4. Discussion

In this study we analyse the influence of emotional exhaustion in chronic diseases, somatic symptoms and social dysfunction, based on the type of activity done as HWs or CHWs.

Among the variables studied, emotional exhaustion achieves the highest average score. These results are consistent with different authors’ findings [13,40], regarding women who carry out long hours of unpaid housework and care tasks. Taking into account the level of overload these women experience due to the tasks they perform [41], emotional exhaustion is predicted [42], and as the level of overload increases, resilience decreases [43]. They generally face this task alone, without the social or family support they need, and are left vulnerable to stress, emotional exhaustion and risk to their health [44]. For the most part, they consider this job to be a role that is assigned to them and they accept it, even sacrificing their personal projects, which favours the development of emotional exhaustion [45]. Social support contributes to a reduction of the effects of stress and overload [46] and works as a protection. 

As for the number of chronic diseases, our results match previous studies that reported on the impact on health according to the number of diseases suffered by family caregivers [47,48] who endure illnesses that could temporarily incapacitate them from performing their duties. However, only 53.4% of participants were considered to have chronic diseases in our study. When grouping them together based on the number of diseases suffered, 37.9% suffered only one, 11.9% suffered two and 3.6% suffered three. One relevant finding was that in 46.6% of participants, their symptoms were not considered chronic diseases, which could explain the variable of the number of diseases obtaining a lower average score than expected. 

Out of the different pathologies (cardiovascular, endocrine-metabolic, mobility-related and others) that they suffered, the highest impact appeared in mobility-related issues (24.4%), matching the results from a study of Muñoz and Barba [49]. Equally, our data show that HWs and CHWs often suffer from pain, a common symptom of several chronic diseases such as fibromyalgia and rheumatoid arthritis, both diseases that provoke swelling, pain and hinder mobility, limiting personal care as well as interest in physical and social interaction [50]. Arthrosis is particularly incapacitating when it affects hips and knees, shoulder pain hinders daily activity and sleep quality [51] and osteoporosis causes intense pain. From this point of view, some studies link the immune system with bone loss in swelling and oestrogen-deficient conditions [52], hence the need for postmenopausal osteoporosis to be checked by several specialists [53]. The decline of functional capacity, provoked by chronic disease, is often experienced with anxiety and/or hopelessness, an attitude that makes it difficult to accept and face up to reality in a manner that makes recovery possible.

Our study confirms the positive and direct link between emotional exhaustion, number of diseases, somatic systems and social dysfunction. In other words, the number of chronic diseases suffered contributes to a higher level of emotional exhaustion, social dysfunction and somatic systems. This could be due to the fact that any chronic disease entails high stress, being worse when more than one disease is suffered from. In this sense, musculoskeletal diseases lead to a significant decrease in women’s physical activity [54], as well as social interaction and communication issues with relatives, stress provoking factors typical of chronic disease [18] that cause emotional changes such as irritability and/or hopelessness. Suffering from more than one disease therefore impacts quality of life from a health point of view [55].

A second finding shows that as emotional exhaustion increases, so do somatic symptoms and social dysfunction. Our results are along the lines of existing literature, as emotional exhaustion is considered to be linked to stress in the workplace [56]. Performing the work activity at home, with little acknowledgement and support, leads to disagreements in family relationships, which in turn affects workplace relationships, as the physical space for roles is shared. The aforementioned work–family conflict gives rise to emotional exhaustion, which in turn worsens the perception of the work–family conflict [57]. A worsening in the level of emotional exhaustion has an impact on the appearance of functional somatic stress, causing symptoms or functional decline that can be linked to the existence of an existential crisis or chronic and life-incapacitating symptoms [17]. The emotional exhaustion experienced leads them to isolate themselves, giving rise to inadequate attitudes of emotional disregard towards the people that they are taking care of. While still looking after them out of a sense of duty. It could however affect the standard of their work [1]. Therefore, as a result of social dysfunction, the tendency to distance themselves from family and friends limits their social interaction [6].

A third finding confirms that an increase in social dysfunction worsens somatic symptoms. Family functionality involves a dynamic process with a constant demand to readapt. Therefore, when dysfunction occurs during the exchange of interpersonal bonds between family members, as a consequence of the failure to adapt to the demands caused by the development of its members in the different stages of their life cycle, it could favour the appearance of conflict [58], largely reflected in somatic symptoms. Therefore, achieving adequate environments based on friendly interpersonal relationships would lead to lower levels of exhaustion and symptoms of anxiety and depression [59].

On the other hand, we can see from the model developed that emotional exhaustion works as a predictive factor of the number of chronic diseases, somatic symptoms and social dysfunction, at different degrees depending on the type of activity performed as a HW or CHW. These results match findings observed in previous research, where emotional exhaustion was considered to be the dimension of burnout that best predicts stress-related effects on health [6]. Caregiving produces a negative effect on the physical and mental health of those women who take on the role of an informal family caregiver [12].

The most common illness amongst CHWs was a finding that was expected, and matched what was reported by authors that talked about an overload increase experienced when taking care of an ill family member [24]. In this sense, the existence of neuropsychiatric symptoms associated with dementia that externalise behavioural disorders such as hallucinations, irritability and depression are considered to be predictive factors of caregivers’ overload [60]. On the other hand, the caregiver’s mental and physical health, as well as the aggressiveness shown by the ill family member, are linked to a risk of suffering from high levels of overload [61]. 

The presence of behavioural disorders such as restlessness/aggressiveness, irritability, abnormal mobility and hallucinations in ill family members with dementia increase caregivers’ level of emotional exhaustion [62], and their quality of life is worse than that of the general population and worsens throughout the progression of the disease [63].

Our findings are along the lines of previous studies, confirming that relationships generated from taking care of others require a constant and intense level of personal/emotional interaction. Although rewarding, this is very stressful for the caregiver, causing very demanding working environments with limited resources [1]. This is why emotional exhaustion has a higher impact on CHWs [10]. 

We can therefore conclude that emotional exhaustion is a predictive factor of the number of diseases, as well as of the somatic symptoms and social dysfunction. Moreover, depending on the type of activity performed by HWs or CHWs, they can be impacted differently at an emotional, social and overall health level. Looking at the previous results, we see that being an Alzheimer patient caregiver, as an added role to that of domestic duties, influences emotional exhaustion, overall health perception and social relationships.

As for the study’s limitations, it is important to point out that the population sampled was not easily accessible, given that they perform their work activity within their own family environment. For this reason, the method we used was convenience sampling. Besides, had the sample been broader, higher numbers might have been obtained in the average scores for emotional exhaustion and number of chronic diseases. Equally, had the study included the Alzheimer patient’s degree of dependence, the CHWs’ results might have been favoured. Finally, the fact that this is a relational study does not allow us to extrapolate cause–effect relationships and the results obtained could be interpreted in a variety of different ways, depending on the perspective of the individual. On the other hand, future studies should analyse the possible intragroup differences of caregiver housewives depending on the level of responsibility for care, social support, whether they have young children or not, whether they have other employees or not and depending on levels of resilience.

## 5. Conclusions

Our results enabled us to know the details of how work and care activities affect HWs and CHWs’ health, with the aim of designing psychosocial and educational interventions to prevent the risks derived from the activity they perform. Interventions should be directed through physical health programmes in order to improve their sleep quality [64], psychosocial programmes for caregivers that provide them with information regarding the disease and how to treat it at each stage, offering emotional support, and implementing strategies to cope, manage stress [65], and reduce anxiety [66] and sedentary behaviour [67], encouraging a healthy alternative to caregiving [68] that includes positive connotation variables, facilitating a change in the perception of their overload and overall health condition, with the objective of providing a higher level of satisfaction and better life qualify indices [69].

## Figures and Tables

**Table 1:** Descriptive statistic and bivariate correlations.

Factor	M	SD	α	1	2	3	4
1. Amount of diseases	0.73	0.82			0.23 **	0.14 *	0.17 *
2. Emotional exhaustion	3.32	1.72	0.82			0.47 **	0.58 **
3. Social dysfunction	0.24	0.29	0.73				0.66 **
4. Somatic symptoms	0.41	0.35	0.75				

** *p* < 0.01; * *p* < 0.05. Note: M = Mean; SD = Standard deviation.
